# Nivolumab plus ipilimumab induced endocrinopathy and acute interstitial nephritis in metastatic sarcomatoid renal-cell carcinoma: A case report and review of literature

**DOI:** 10.3389/fimmu.2022.993622

**Published:** 2022-08-16

**Authors:** Christopher Hino, Kevin Nishino, Bryan Pham, Won Jin Jeon, Michael Nguyen, Huynh Cao

**Affiliations:** ^1^ Department of Internal Medicine, Department of Medicine, Loma Linda University, Loma Linda, CA, United States; ^2^ Department of Oncology/Hematology, Department of Medicine, Loma Linda University, Loma Linda, CA, United States

**Keywords:** Sarcomatoid renal cell carcinoma (SRCC), nivolumab (PubChem SID: 178103907), ipilimumab (PubChem SID: 178103470), immune related adverse events, adrenal insufficiency, acute interstitial nephritis (AIN), hypothyroidism, diabetes mellitus

## Abstract

The prognosis of sarcomatoid renal cell carcinoma has changed dramatically with the emergence of immune checkpoint inhibitors. Notably the use of nivolumab and ipilimumab combination therapy has demonstrated promising durable therapeutic response for patients with treatment-naïve sarcomatoid renal-cell carcinoma. We present a case of 45-year-old man with a history of metastatic sarcomatoid renal cell carcinoma treated with nivolumab plus ipilimumab who developed type 1 diabetes mellitus, adrenal insufficiency, thyroiditis/hypothyroidism, and acute interstitial nephritis as a result of immunotherapy.

## Introduction

Sarcomatoid renal-cell carcinoma (sRCC) is a rare and highly aggressive form of dedifferentiated renal carcinoma that portends an exceptionally poor prognosis (1). Approximately 60-80% of patients with sRCC present with advanced or metastatic disease at the time of diagnosis, and are known to have a poor response to traditional chemotherapy. Up until recently, the median overall survival for sRCC was approximately 6-13 months (2, 3). However, the emergence of combination immune checkpoint inhibitors (ICIs) with nivolumab (NIVO; a programmed death 1 immune checkpoint inhibitor antibody) plus ipilimumab (IPI; a cytotoxic T-lymphocyte antigen 4 antibody) as first line therapy for advanced clear cell RCC has radically improved the survival prognosis of advanced RCC (4). More recently *post-hoc* analysis of the Phase III CHECKMATE 214 trial demonstrated promising efficacy and prolonged survival in patients with sRCC who received ipilimumab and nivolumab vs sunitinib- with a median OS of 31.2 months vs 13.6 and ORR fo 57% vs 19% (5).

Immune related adverse events (irAEs) associated with ICIs have resulted in significant morbidity through autoimmune-like toxicities (6). Although the occurrence of irAEs has been well documented in other solid tumors, the safety profile of ipilimumab and nivolumab for use in RCC has yet to be fully elucidated. Here we report a case of a 45-year-old man with metastatic sRCC who was treated with NIVO+IPI and developed several uncommon irAEs including type 1 diabetes mellitus, adrenal insufficiency, thyroiditis/hypothyroidism, and acute kidney injury likely from acute interstitial nephritis.

## Case presentation

**Figure 1:**
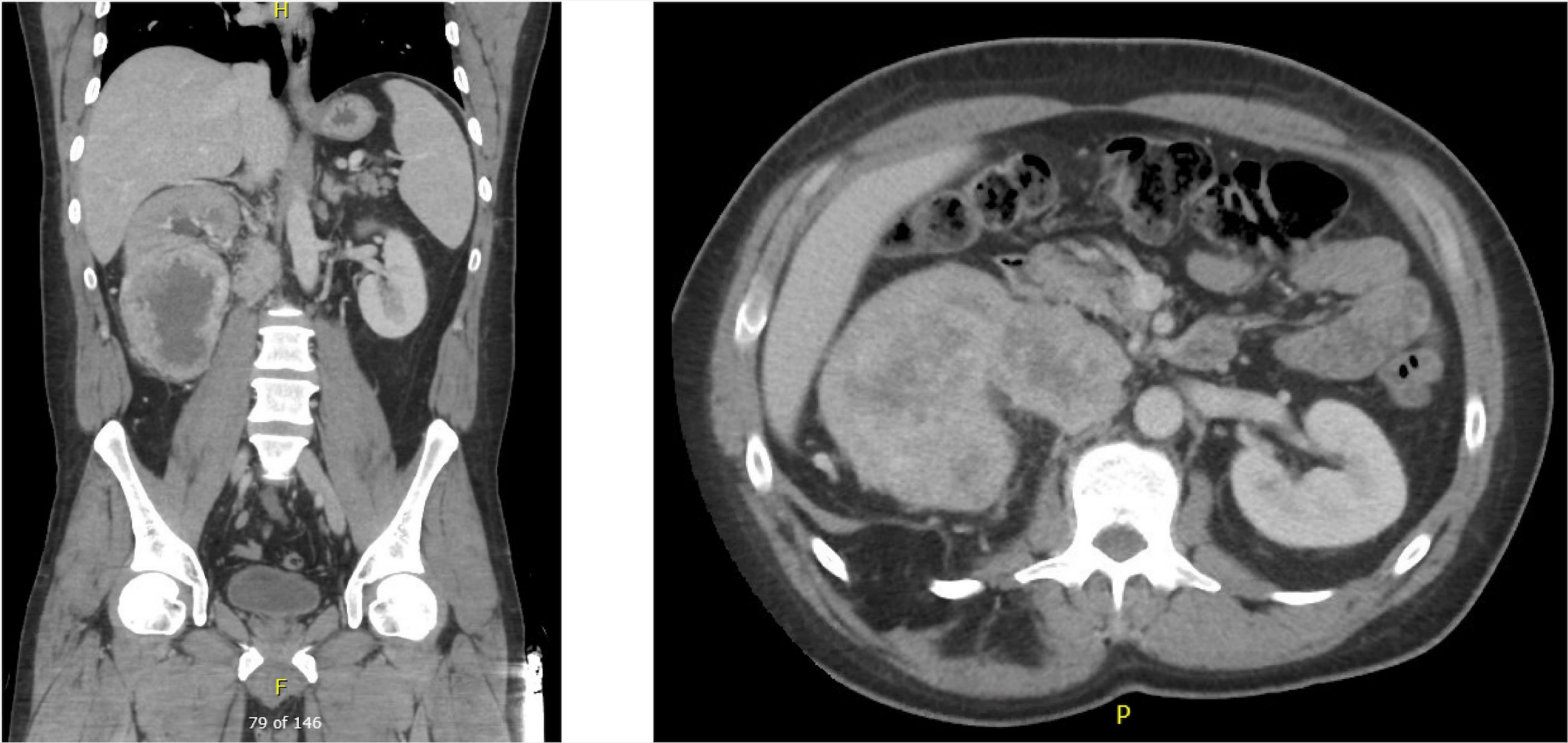
Pre-nephrectomy CT showing multiple right renal masses with tumor thrombus extending into the right renal vein and around the IVC.

A 45 year-old male with an unremarkable past medical history and no known history of autoimmune disease, diabetes, adrenal insufficiency, or chronic kidney disease presented with severe abdominal pain, right sided lower back pain, fatigue, and night sweats was found to have a 12 x 11 x 8 cm right renal mass with tumor thrombus extending into the right renal vein and around the inferior vena cava (IVC) (**Figure 1**). He subsequently underwent right radical nephrectomy with retroperitoneal lymphadenectomy and partial cavectomy with bovine patch onto the cava for IVC repair. Pathology of the renal mass confirmed a diagnosis of clear cell renal carcinoma with 60% sarcomatoid differentiation and focal rhabdoid features. Sarcomatoid renal cell carcinoma was also confirmed by pathology in 3 out of 4 retrocaval lymph nodes and 1 out of 6 interaortocaval lymph nodes. In addition, tumor adherent to and excised from the IVC was positive for sarcomatoid renal cell carcinoma. Further work-up with a CT chest showed a 6 mm pulmonary nodule in the right middle lobe suspicious for metastasis. However, MRI brain showed no signs of metastasis. The patient was clinically staged T4N1M1 with G4 histologic features.

Given sarcomatoid subtype and patient’s advanced disease, the patient was started on NIVO+IPI combination therapy. Within 1 week of initiation of NIVO + IPI therapy, the patient reported dark stools and was found to be anemic with Hgb of 5.1 g/dl. Further investigation revealed a 3 cm bleeding duodenal mass that was subsequently treated with coil and plug embolization of the gastroduodenal artery. A biopsy of the duodenal mass was not pursued given patient’s high risk for rebleeding. Following hemodynamic stabilization, the patient was continued on NIVO + IPI.

Two months into treatment, the patient was found to have TSH of 0.074 μIU/mL with free T4 level of 2.89 pmol/L. He was subsequently referred to endocrinology for concern for immunotherapy induced thyroiditis. Approximately 2 months later, the patient’s TSH increased to 63.57 μIU/mL and free T4 level decreased to 0.31 pmol/L. Due to concern for immunotherapy induced hypothyroidism, the decision was made to start levothyroxine. Follow up CT with contrast of the chest, abdomen, and pelvis showed decreased size of the duodenum and inferior vena cava masses. The 6 mm nodular mass seen in the right middle lobe on previous scan was no longer visualized.

Four months after initiation of NIVO + IPI therapy, the patient was found to have an acute kidney injury (AKI) with serum creatinine (SCr) increasing to 1.7 mg/dL from the patient’s baseline SCr of 1.2 mg/dL. His blood urea nitrogen (BUN) was 22 mg/dL with a BUN/creatinine ratio (BCR) of 12.9. A urinalysis at the time of AKI and 4 months prior to initiation of NIVO+IPI therapy was unremarkable for proteinuria, hematuria, pyuria, or abnormal urine sediment. Furthermore, renal ultrasonography showed no evidence for hydronephrosis or intraparenchymal disease. Microscopic evaluation for urine eosinophils showed only 0-1/hpf eosinophils/hpf. He had also been found to have to have serum eosinophil of 10.8%. Given absence of proteinuria, abnormal urine sediments, but presence of eosinophilia and eosinophiluria we had suspected mild (grade 1) immunotherapy related acute interstitial nephritis, especially in the setting of multiorgan irAE. However we could not exclude contrast induced nephropathy given recent IV contrast for imaging. Therefore, immunotherapy was held and the patient was fluid resuscitated and started on prednisone 1 mg/kg. The patient was treated with fluid resuscitation and a two week course of high-dose prednisone resulting in partial improvement in renal function with a Cr to 1.4 mg/dL. Preventative fluid resuscitation was given on a weekly basis while the patient was continued on treatment to prevent worsening of kidney function. To date, renal biopsy has not been pursued.

Eight months after starting NIVO + IPI therapy, the patient presented to hospital for one week of fatigue, worsening weakness, nausea, and blurry vision. However he denied concomitant rashes, pruritus, diarrhea or other ophthalmologic symptoms. He was found to have a blood sugar level of 667 mg/dL and hemoglobin A1c level of 7.6 on admission; thus a new diagnosis of diabetes mellitus (DM) was made. Laboratory investigation showed negative anti-glutamic acid decarboxylase antibodies, negative insulin antibodies, and low C peptide level of 0.5 ng/ml. Based on these findings, we suspect that the patient likely developed autoimmune type 1 diabetes mellitus from nivolumab therapy. He was consequently started on a daily metformin and insulin regimen.

Given our concerns for the potential side effects from immunotherapy, a decision was made to discontinue nivolumab and start sunitinib. However, the patient developed excessive fatigue, joint pain, mouth pain, thrombocytopenia and worsening Cr level to 1.9 mg/dL while on sunitinib. Sunitinib was subsequently discontinued after approximately 3 weeks of therapy, and the patient was transitioned back to nivolumab monotherapy.

Despite adequate treatment for his hypothyroidism, the patient reported progressive fatigue and memory loss for two months around the same time as he had been restarted on NIVO. Further work-up revealed a morning cortisol level of 0.3 ug/dL with an ACTH level of 3.8 pg/mL. The low ACTH and cortisol level supported the diagnosis of secondary adrenal insufficiency likely due to Nivolumab induced hypophysitis. The patient was started on treatment with hydrocortisone 25 mg BID with marked clinical improvement. The patient was resumed on immunotherapy treatment. PET scan obtained 11 months after NIVO-IPI therapy initiation showed no evidence of tumor recurrence or metastatic disease.

## Discussion

**Figure 2:**
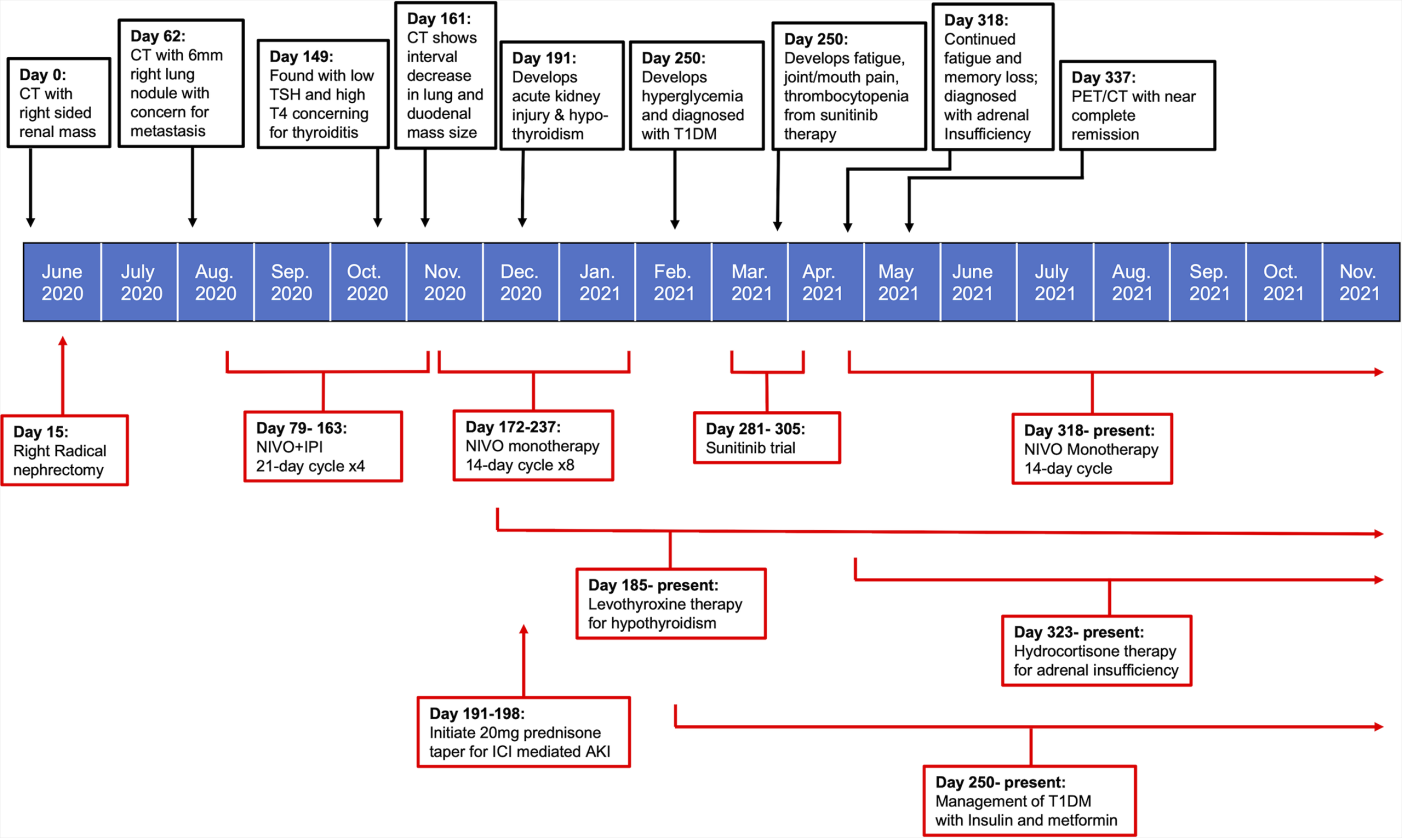
Timeline summarizing patient’s treatment course in relation to onset and management of irAEs.

Immune checkpoint molecules are crucial to the maintenance of self-tolerance and the modulation of immune mediated injury, but also play a key role in tumor immune escape (7). With the recent paradigm shift in the use of ICIs for sarcomatoid RCC, we have likewise seen both durable therapeutic responses and a concomitant change in the type of side effects observed with treatment (3). The CHECKMATE 214 trial was the first major study to document the prevalence of irAEs with NIVO + IPI combined therapy for advanced RCC (5). The reported frequency of irAEs of any grade in RCC patients treated with NIVO+IPI was found to be 81%; with dermatologic (49.8%), endocrine (33%), and gastrointestinal (29.6%) irAEs being among the most commonly reported. Treatment- related adverse events led to discontinuation in 22% of study participants, demonstrating both the severity of irAEs and the need for improved management strategies in patients with intolerable side effects (8). The case presented here highlights an exceptionally uncommon spectrum of multiorgan irAEs observed with NIVO+IPI therapy in sRCC, and the subsequent management of these multiorgan side effects (**Figure 2**).

ICI-induced endocrinopathies, such as adrenal insufficiency and fulminant type 1 diabetes mellitus are extremely rare and potentially life-threatening conditions that were observed in the patient discussed above (9, 10). The reported incidence of adrenal insufficiency with NIVO+IPI therapy ranges between 0.7- 29%, however the known incidence in RCC is reported between 4.7-10.2% (8–13). This discrepancy is most likely due to the limited sample size of current studies and infrequent recognition of adrenal insufficiency in these patients. In our case, the vague presenting symptoms of progressive fatigue and low energy prompted early evaluation and treatment for adrenal insufficiency. While use of high dose corticosteroids may temporarily alleviate symptoms, ICI-induced adrenal insufficiency is rarely reversible and often requires long term glucocorticoid replacement (12).

The development of type I diabetes mellitus (T1DM) is an even more rare manifestation of ICIs, with an estimated incidence between 0.2 and 1.4% (12, 14–18). Previous meta- analysis has shown that the onset of type 1 diabetes mellitus often occurs within the first 3 months of starting therapy. Remarkably only half of previously reported cases were found to have detectable type 1 diabetes-associated antibodies at presentation. By comparison ~90% of people with prototypical childhood-onset type 1 diabetes have one or more antibodies at diagnosis (17). Our patient was not found with anti-GAD or anti-islet antibodies at the time of presentation, however his low C-peptide, insulin deficiency, and unexplained hyperglycemia prompted treatment with exogenous insulin therapy for T1DM. The management of diabetes is challenging in the present case, especially given that use of steroids was indicated for management of the patient’s other presenting irAEs, but also appears to be ineffective in reversing beta cell dysfunction and may even worsen insulin resistance (14).

Thyroid dysfunction is the most frequently cited ICI- endocrinopathy. It can occur within 3 weeks to 10 months following ICI therapy, with a frequency between 5.2- 28% with combination ICI therapy. While Anti-PD1/PDL-1 agents are predominantly associated with thyroid irAEs, recent studies suggest that patients receiving combination ICI therapies are at greater risk for thyroid dysfunction (9, 10). The patient described above developed thyroiditis which eventually progressed to overt hypothyroidism. In most cases, hypothyroidism remains permanent and requires long-term levothyroxine replacement as seen in this patient (10).

The incidence rate of ICI-induced acute interstitial nephritis in metastatic renal cell carcinoma is estimated to be 1.7% (19, 20). Given the acute rise in creatinine, eosinophilia, eosinophiluria we suspect that our patient developed ICI-induced acute interstitial nephritis. However no kidney biopsy was obtained to definitively confirm the diagnosis. The difficulty in diagnosing AIN in the present case was complicated by recent contrast use and development of uncontrolled DM, however recovery of renal function following ICI discontinuation and steroid administration points to the likely diagnosis of AIN.

While guidelines have been established for the management of common irAEs, the current case demonstrates 1) the need to establish strategies for managing multiorgan irAEs and 2) the importance of monitoring for uncommon but potentially life-threatening manifestations. Here we show that despite a promising response to therapy, the management of multiple concurrent irAEs may complicate and potentially interfere with the ability to continue ICIs in the future. This raises a particularly relevant and interesting dilemma that has not yet fully been addressed in the literature: Should patients with good initial response to ICIs continue/restart therapy in the setting of moderate-severe irAEs?

Accumulating evidence suggests that the increased occurrence of irAEs is associated with durable therapeutic responses with ICIs (20–29). This association has also been independently observed in mRCC (30–35). The current case adds support to the literature, suggesting that patients with severe multiorgan irAEs may have improved response to ICI therapy. Based on these observations, it is reasonable to hypothesize that irAEs are a byproduct of a hyper-responsive immune state that simultaneously promotes anti-tumor immunity and autoimmunity.

Previous studies have observed that patients frequently developed eosinophilia prior to the onset of ICI induced adrenal insufficiency and that eosinophilia may be an independent predictor for a favorable response to therapy (13, 36–40). However it should be noted that eosinophilia can also be an early sign of adrenal insufficiency independent of ICI therapy (41). ICI-induced acute interstitial nephritis, which is also associated with eosinophilia, has additionally been implicated in predicting a favorable response to ICIs in RCC (19). The current case corroborates these findings, and may point towards a shared uncharacterized mechanism between irEAs and effective ICI response.

The similarity between irAEs observed in our patient and the polyendocrinopathy characterized in IPEX syndrome may allude to the critical role of regulatory T cells (Tregs) in mediating both the clinical efficacy and irAEs associated with ICI therapy. Treg dysfunction through mutation in its master transcription factor, FOXP3, drives the immune dysregulation observed in IPEX (immunodysregulation polyendocrinopathy enteropathy X-linked) syndrome (42). It would thus seem reasonable that a blockade of immune checkpoints expressed on intratumoral Tregs such as PD-1 and CTLA-4 could promote an IPEX-like syndrome, as observed in the present case.

Current guidelines recommend permanent discontinuation of ICIs that induce severe grade 4 irAEs (6, 43). However, recent studies have suggested that it may be conceivable to rechallenge patients with the same ICI after resolution of symptoms (44). In support of this, a recent large retrospective cohort study observed only a 28.8% irAE recurrence rate following rechallenge with the same inciting ICI (45). In the present case, we did not observe additional irAE following rechallenge with NIVO monotherapy. This strengthens the possibility that ICI rechallenge may be beneficial in sRCC patients who respond well to initial therapy.

## Conclusions

In conclusion, ICI- associated endocrinopathies and acute interstitial nephritis are rare irAEs, but are important to recognize with NIVO+IPI therapy in sRCC. Clinicians should have a high index of suspicion for these uncommon manifestations, which should prompt urgent evaluation, discontinuation of therapy, and initiation of appropriate irAE therapy when indicated. Future studies should determine the most appropriate management strategy for patients with sRCC who develop severe irAEs despite good response to ICI therapy.

## Data availability statement

The original contributions presented in the study are included in the article/Supplementary Material. Further inquiries can be directed to the corresponding author.

## Ethics statement

Ethical review and approval was not required for the study on human participants in accordance with the local legislation and institutional requirements. The patients/participants provided their written informed consent to participate in this study. Written informed consent was obtained from the individual(s) for the publication of any potentially identifiable images or data included in this article.

## Author contributions

CH, KN, BP, WJ wrote and edited the manuscript. MN and HC contributed to the diagnosis and treatment of case, and revised the paper. All authors read, approved the submitted version, and agreed to be accountable for all aspects of the research in ensuring the accuracy of this study. All authors have given consent to the publication of this manuscript.

## Conflict of interest

The authors declare that the research was conducted in the absence of any commercial or financial relationships that could be construed as a potential conflict of interest.

## Publisher’s note

All claims expressed in this article are solely those of the authors and do not necessarily represent those of their affiliated organizations, or those of the publisher, the editors and the reviewers. Any product that may be evaluated in this article, or claim that may be made by its manufacturer, is not guaranteed or endorsed by the publisher.
